# 

**Published:** 1867-08

**Authors:** 


### NOTICE TO DENTISTS.

The Mad River Valley Dental Association will hold their next Quarterly Meeting in the city of Springfield, O., convening at the Willis House, the first Tuesday in September—(the 3rd day)—as early as can be reached by the trains.

Dear Brethren, we have a splendid programme for your entertainment. We most cordially insist upon your coming, and participation throughout the session.

The Essayists are, Prof. G. Watt, M. D., D. D. S. Subject—Nitrous Oxide.

A. A. Blount, M. D. Subject—The relation existing between the Medical and Dental Practitioner.

Dr. A. Berry, Dr. Campbell, Dr. M. M. Oldham,—Subjects optional.

The subjects for discussion are, 1st. Dental Pathology—to be opened by Dr. Watt.

2nd. Physical Conditions of the Teeth—Dr. Taft.

3rd. What conditions modify the effects of operations on the Teeth—Dr. G. W. Keeley.

4th. What treatment do these conditions require?—to be opened by J. H. Paine.

N. W. Williams, President.

J. H. Paine, Cor. Sec'y.

Middletown, O., Aug. 13, 1867.

### CONTENTS.

### OF THE WEST.

*Issued Monthly, at $3 a Year in Advance.*

DEVOTED TO THE INTERESTS OF THE DENTAL PROFESSION.

EDITED BY J. TAFT AND G. WATT.

The volume commences in January and closes in December.

The Register being issued monthly is always fresh, therefore indispensable to the practitioner who desires to keep posted up with the new inventions and improvements which are daily developed. Neither expense nor effort will be spared to give value and variety to its contents. Articles will be illustrated with well executed engravings whenever they will add to the interest or assist in explanation.

Its extensive circulation and frequency of issue makes it one of the BEST DENTAL ADVERTISING MEDIUMS in the United States.

RATES OF ADVERTISING.

One page, 1 year, $50. Half page, 1 year, $30. Quarter page or card, 1 year $20.

All advertising bills payable in advance, or at furthest after the issue of the first number containing the advertisement. All transient advertisements to be paid for in advance.

☞ CONTRIBUTIONS SOLICITED.

Address, J. TAFT, or "DENTAL REGISTER," Cincinnati, Ohio.

Business Communications to

J. TAFT, Publisher,
No. 238 Fourth St., between Plum and Central Avenue.

